# Going Beyond the “Synthetic Method”: New Paradigms Cross-Fertilizing Robotics and Cognitive Neuroscience

**DOI:** 10.3389/fpsyg.2022.819042

**Published:** 2022-06-03

**Authors:** Edoardo Datteri, Thierry Chaminade, Donato Romano

**Affiliations:** ^1^RobotiCSS Lab, Laboratory of Robotics for the Cognitive and Social Sciences, Department of Human Sciences for Education, University of Milano-Bicocca, Milan, Italy; ^2^Aix-Marseille Université, Institut de Neurosciences de la Timone, UMR 7289, CNRS, Marseille, France; ^3^Sant’Anna School of Advanced Studies, The BioRobotics Institute, Pisa, Italy; ^4^Department of Excellence in Robotics and AI, Sant’Anna School of Advanced Studies, Pisa, Italy

**Keywords:** robotics, biomimetics, ethorobotics, biorobotics, philosophy of science

## Abstract

In so-called ethorobotics and robot-supported social cognitive neurosciences, robots are used as scientific tools to study animal behavior and cognition. Building on previous epistemological analyses of biorobotics, in this article it is argued that these two research fields, widely differing from one another in the kinds of robots involved and in the research questions addressed, share a common methodology, which significantly differs from the “synthetic method” that, until recently, dominated biorobotics. The methodological novelty of this strategy, the research opportunities that it opens, and the theoretical and technological challenges that it gives rise to, will be discussed with reference to the peculiarities of the two research fields. Some broad methodological issues related to the generalization of results concerning robot-animal interaction to theoretical conclusions on animal-animal interaction will be identified and discussed.

## Introduction

Throughout history, machines endowed with sensing and actuating devices—now called robots—have been often involved in the development of philosophical and empirical theories on the form and mechanisms of animal behavior and cognition (Tamborini, 2021, 2022). Their role has occasionally been *inspirational*. According to Riskin (2016), for example, Descartes’ mechanistic reconstruction of the animal body may have been inspired by his visits to the garden of Saint-Germain-en-Laye, populated by hydraulic, responsive automata representing mythological characters. In this and other cases, automata built for purposes other than scientific research (e.g., entertainment) have been interpreted as constructive proofs that certain aspects of animal behavior can be reproduced mechanically, thus inspiring mechanistic conceptions of life and animal behavior.

At least since the beginning of the twentieth century (but see Mantovani, 2020, for a historical precedent), robots have occasionally played a different role in the study of behavior and cognition. In cybernetics (Cordeschi, 2002) and, more recently, biorobotics (Webb, 2001; Webb and Consi, 2001; Datteri, 2017; Gravish and Lauder, 2018), robots are deliberately built for *experimental* purposes: they are used as experimental tools for the discovery and testing of theories on animal behavior and cognition. The term “biorobotics” will be used here in a very broad sense, as referring to the experimental use of robots to discover and test theories in the life and social sciences. From a methodological point of view, it can be argued that contemporary biorobotics can be ideally split into (at least) two broad branches. They differ from one another in at least three features, which can be introduced by the following questions:

– Is the robot a model of the system under investigation?
– Is the robot used for surrogative reasoning?
– Does the study conform to the “synthetic method”?

The content of these questions will be clarified in the ensuing sections. It will be argued here that the answer to these questions is affirmative for many contemporary biorobotic studies, which will be called *classical* here. Other biorobotic studies, which will be called here *interactive*, admit of a negative answer. As stressed later, clarifying the methodological distinction between these two branches of biorobotics is worth the effort for some reasons. First, a methodological analysis of biorobotics may reveal the methodological diversity of the field. Second, it may help one identify the theoretical and epistemological assumptions on which biorobotic results rest, depending on the structure of the experimental procedure. Third, it can lead one to establish that different subfields of biorobotics display methodological commonalities, as it will be argued in the ensuing analysis of ethorobotics and social cognitive neuroscience.

### Classical Biorobotics

The methodological characteristics of classical biorobotics will be explored here starting from the three questions introduced above and using a study on bat echolocation (Bou Mansour et al., 2019) as an example of this branch of biorobotics.

#### Is the Robot a Model of the System Under Investigation?

**FIGURE 1:**
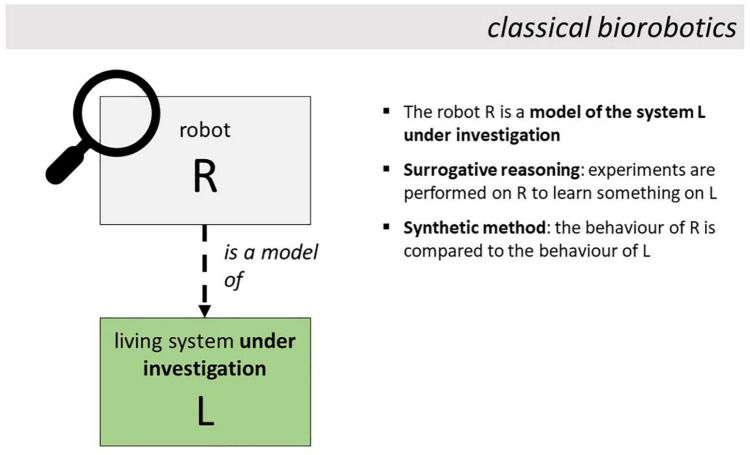
In classical biorobotics, the robot is a model of the living system under investigation and is used to perform surrogative reasoning on it. Experiments are performed on R (as represented by the position of the lens). The behavior of the robot is compared to the behavior of the system L under investigation.

In classical biorobotics, the robot (R from now on, see Figure 1) is regarded as a model of the living system L under investigation. For example, in the study on bat echolocation described in Bou Mansour et al. (2019), the robot is regarded by the authors as a model of a bat. And, arguably, the same can be said of all the biorobotic studies reviewed in Gravish and Lauder (2018). The nature of the relationship holding between two concrete systems, when the first is regarded as a model of the second one, has been extensively discussed in the philosophical literature. What makes a concrete system, R in our case, a model of another concrete system L? Here it will be assumed, according to the so-called *inferential* account proposed by Suárez (2004), that a robot R is a model of living system L if (a) the experimenter stipulates that R represents L, and (b) R, by virtue of its characteristics, may in principle enable one to make inferences about L (e.g., about some aspects of L’s behavior or internal mechanism). Both conditions are satisfied in the bat study. The authors stipulate that the robotic bat represents a bat, and the robot, by virtue of its characteristics, may enable the authors to make inferences about the behavior of bats—specifically, about the mechanisms of obstacle avoidance in real-life bats. It is claimed here that all classical biorobots can be regarded as models of the system under investigation in this sense (see Frigg and Nguyen, 2017 for other definitions of the concept of scientific model). Note that, as discussed later, robots are models of living organisms in interactive biorobotics too, with a crucial difference: whereas, in classical biorobotics, the robot is a model of the living system *under investigation*, in interactive biorobotics the robot is a model of a system *other than* the system under investigation.

#### Is the Robot Used for Surrogative Reasoning?

In classical biorobotics, the robot R is used as an experimental surrogate of the system L under investigation (which is also the system of which R is a model). By performing experiments on the surrogate, one acquires knowledge about L. The knowledge obtained in classical biorobotics can be of two different kinds. In so-called model-oriented studies (Datteri, 2021a), experiments on the surrogate enable one to discover the mechanism governing some aspects of L’s behavior. In so-called prediction-oriented studies, experiments on the surrogate enable one to predict the behavior that L would generate in some circumstances. The position of the lens in Figure 1 represents the fact that, in classical biorobotics, the experiments are performed on R, meaning that R’s behavior is the subject of analysis. The term “surrogative reasoning” was coined by Swoyer (1991) and is now widely used in the philosophical literature to refer to the primary role of scientific models: they enable surrogative reasoning, meaning that they enable one to reason about the modeled system using a surrogate of it. Surrogative reasoning characterizes classical biorobotics which is, in this respect, a typical form of model-based science (Magnani and Bertolotti, 2017). As discussed below, in interactive biorobotics the robot is used for purposes other than surrogative reasoning.

#### Does the Study Conform to the “Synthetic Method”?

Classical biorobotics, and more specifically model-oriented classical biorobotic studies, typically adopt the so-called “synthetic method” which has also been dubbed “understanding by building approach” (Pfeifer et al., 2008) and frequently adopted in cognitively-driven Artificial Intelligence (Newell and Simon, 1972) and neuroscience (Kawato, 2008). The synthetic method, as thoroughly discussed in Cordeschi (2002), is a particular form of surrogative reasoning (meaning that not all surrogative reasoning, in robotics and beyond, conforms to the synthetic method). The synthetic method is an experimental strategy, and a form of scientific reasoning, in which the robotic model R is used for a particular purpose, i.e., *to discover the mechanism governing the behavior of the modeled system L* (whereas, as anticipated, other forms of biorobotics aim at predicting L’s behavior, which is clearly different from discovering the mechanism that governs it). It is a comparative approach. The robot (R) implements a mechanism (*Mech*) that is hypothesized to produce some aspects of the behavior of L. For example, in the bat study, the robot implements a mechanism based on active acoustic gaze scanning and detection of interaural level difference which is hypothesized to produce good echolocation in bats (echolocation refers to bats’ capacity to localize and avoid obstacles relying on the analysis of echos of ultrasound emitted by the animal). The behavior of R and L is compared in particular experimental conditions. Behavioral matches or mismatches are taken as evidence to accept or refute the hypothesis that *Mech* governs the behavior of L as well. For example, in the bat study, R did not reach good levels of echolocation, and this led the authors to refute the hypothesis that bats implement *Mech* as it stands. What matters for the present study is that classical biorobotic studies typically involve experimental comparisons between the behavior of the robot R and the behavior of the modeled system L. A comparison which, as claimed below, is not made in interactive biorobotics.

### Interactive Biorobotics

Recent years have witnessed the emergence of methodologies for the use of robots to study animal cognition and behavior which differ from classical biorobotics. Notwithstanding their diversity, they all involve forms of *interaction* between robotic devices and living systems. One such development is taking place in the field of social cognitive neuroscience. Social cognitive neuroscience (SCN from now on) is distinctively concerned with the study of the cognitive and neurophysiological mechanisms underlying social behavior (Liebermann, 2010). Quite recently, in this field of research, humanoid robots interacting with humans have been used “as a new type of ‘stimuli’ in psychological experiments” (Chaminade and Cheng, 2009; Wykowska, 2020). They have been claimed to “provide insightful information regarding social cognitive mechanisms in the human brain” (Wykowska et al., 2016). The subfield of SCN using robots for this purpose (Chaminade and Kawato, 2011) will be referred to as rSCN from now on (the “r” standing for “robot-supported”). This use of robots characterizes so-called “android science” too (MacDorman and Ishiguro, 2006), as discussed later. Another field in which such development is taking place is so-called ethorobotics (Romano et al., 2019). In ethorobotics, animal-like robots, interacting with real-life animals in more or less ecological contexts, have been claimed to “have the potential to revolutionize the study of social behavior” (Krause et al., 2011) and to constitute “a novel method for studying collective animal behavior” (Faria et al., 2010).

In these two research fields, robots are used to theorize on animal cognition and behavior. To be sure, these fields significantly differ from one another in the target system (respectively, human beings and non-human animals), in the typical characteristics of the robots involved (respectively, humanoid and animaloid robots), and broadly speaking in the research questions addressed. However, at some level of analysis, it can be argued that these two research fields share the same methodological approach.

**FIGURE 2:**
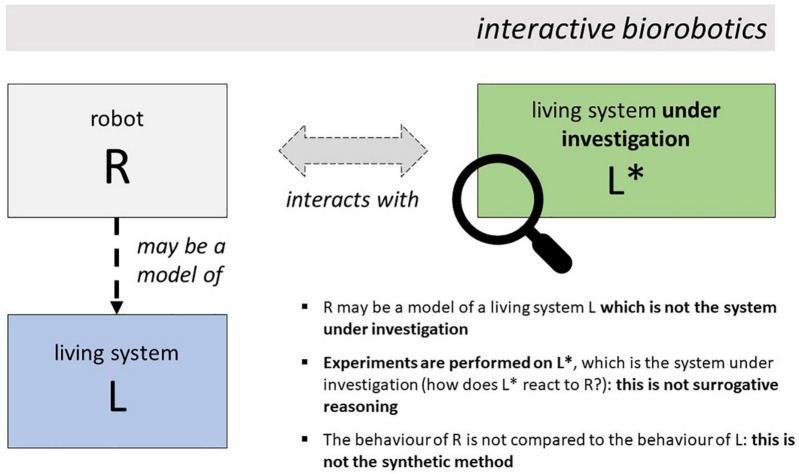
In interactive biorobotics, the robot R may be a model of a living system L. However, it is not used to perform surrogative reasoning on it. Rather, it is used to study the behavior of another system, L*, with which it interacts in suitable experimental conditions.

Indeed, in rSCN and ethorobotics, a robot R *interacts with* the living system L* under investigation (see Figure 2). This is the first point of departure from classical biorobotics, where the robot *does not interact* with the system under investigation (there called L). Another striking difference is that the subject of experimental analysis is not the robot, as in classical biorobotics (where one is interested in the behavior produced by the robot in controlled experimental settings, and in whether it can reproduce L’s behavior), but the system under investigation: in the interactive approaches discussed here, one analyses the reactions of L* to the stimulations delivered by the robot, as represented by the position of the lens in Figure 2. Other finer-grained differences emerge in connection with the three features of classical biorobotics identified before (see Table 1 for a summary).

**TABLE 1:** Methodological differences between classical and interactive biorobotics.

	Is the robot a model of the system under investigation?	Is the robot used for surrogative reasoning?	Does the study conform to the “synthetic method”?
Classical biorobotics	Yes	Yes	Yes
Interactive biorobotics	No	No	No

#### Is the Robot a Model of the System Under Investigation?

In some cases, the robot R is a model of a biological system L. For example, in some rSCN and ethorobotic studies, the robot is a model of a human being or of a non-human animal. However, unlike biorobotics, this does not need to be the case. In rSCN and ethorobotics, one may decide to observe L*’s reactions to stimuli coming from robots which do not model any living system (for an example, see Blanke et al., 2014). Moreover, while in classical biorobotics the robot R is a model of the system under investigation (e.g., of a bat in the aforementioned study), in rSCN and ethorobotics the robot is not a model of the system under investigation, which is L* in Figure 2. This consideration has some implications on whether rSCN and ethorobotics can be said to involve forms of surrogative reasoning, as discussed below.

#### Is the Robot Used for Surrogative Reasoning?

In rSCN and ethorobotics, R is not used to theorize on the system of which it is a model (i.e., on L in Figure 2). Rather, it is used to study the system L* *with which it interacts*. Thus, even when R is a model of a living system L, L is not the system under investigation, or, equivalently, R is not used to perform surrogative reasoning on the modeled system. This is an important point of departure from classical biorobotics and, more generally, from model-based science as traditionally conceived (Magnani and Bertolotti, 2017), which is based on the use of surrogates to reason about the modeled system. In the interactive approaches discussed here, the system L which is modeled by the robot does not coincide with the system L* which is the focus of investigation (Datteri, 2020, 2021a).

#### Does the Study Conform to the “Synthetic Method”?

The claim that social robotics and ethorobotics do not adopt the “synthetic method” characterizing classical biorobotics and AI-based studies of cognition partially follows from the observations made above. The goal of these studies is not to discover the mechanism governing the system L modeled by the robot. Their goal is to study the behavior of the system L* interacting with the robot. Also note that, unlike the synthetic method, the approach described here does not involve any comparison between the behavior of the robot R and the behavior of the modeled system L. Neither they involve a comparison between the behavior of R and the behavior of the system L* under investigation: the goal is not to find out whether R can reproduce L*’s behavior, but to study L*’s reactions to the presence and characteristics of R. Table 1 summarizes the differences between classical and interactive biorobotics discussed here, and Table 2 provides definitions of some key terms used in this article.

**TABLE 2:** Definitions of the key terms used in the text.

Term	Definition
Model	The term is used here to refer to a concrete system that stands for, or represents, another concrete system. See Frigg and Nguyen (2017) for an analysis of the conditions under which a system R can be regarded as a model of another system L. The so-called inferential conception of scientific modeling (Suárez, 2004) is presupposed here.
System under investigation	The system studied in a piece of research. In biorobotics, the system under investigation is a living system.
Modeled system	If R is a model of living system L, L is the modeled system. In classical biorobotics, L is the modeled system and the system under investigation. In interactive biorobotics, L is not the system under investigation.
Surrogative reasoning	The (possibly experimental) use of a model R to draw inferences about the modeled system (Swoyer, 1991).
Synthetic method	A comparative strategy that can be adopted to discover the mechanism governing the behavior of a living system L. It involves building a robot R that implements the mechanism which is hypothesized to govern L’s behavior, and comparing the behaviors of R and L. It is widely adopted in classical biorobotics. See Cordeschi (2002) for an extensive discussion.
Biorobotics	The term refers to the experimental use of robots to discover and test theories in the life and social sciences.
Social cognitive neuroscience (SCN)	Is a research field concerned with the study of the cognitive and neurophysiological mechanisms underlying social behavior (Liebermann, 2010).
Robot-supported social cognitive neuroscience (rSCN)	Is a subfield of SCN in which robots are used to study the cognitive and neural mechanisms underlying social behavior.
Ethorobotics	Is a research field characterized by the use of robots to study and/or modulate animal behavior (Krause et al., 2011; Romano and Stefanini, 2021c).

### Biorobotics and Philosophy of Science

As pointed out before, comparing classical with interactive biorobotics is worth the effort for a number of reasons. It may reveal the methodological diversity of biorobotics. Robots can be used to study cognition and behavior in many ways, and this diversity can be appreciated by raising one’s sight over the level of the individual studies and adopting a more general, methodological perspective. Reconstructing the methodologies adopted in the field may also end up uncovering potentially fruitful experimental strategies that have rarely or never been attempted so far (an example being the simulation-interactive methodology discussed in Datteri (2021b). It may also reveal methodological issues that distinctively arise in one or the other branch of biorobotics. A few examples will be briefly discussed here.

Interactive biorobotic studies involve inferential steps that are not carried out in classical biorobotics (and vice versa). One of them has been discussed in Datteri (2020). In interactive studies, the behavior of the living system under investigation (L* in Figure 2) is analyzed during interaction with robot R. As such, the experimental results *prima facie* concern L*’s reactions to a *robot*. However, many interactive biorobotic studies aim at generalizing this kind of results to obtain theoretical conclusions on the social dynamics occurring within groups of living systems. In these cases, experimental results concerning the relationship between L* and R (e.g., between a human and a humanoid robot) are taken as a basis to draw theoretical conclusions about the relationship between L* and other, possibly conspecific, living systems (e.g., between humans). This inferential step, when carried out, clearly needs justification: one cannot simply take for granted that L*’s reactions to R will be the same as those that L* would display, in similar circumstances, interacting with another living system. How this inference can be justified is a question that falls out of the scope of this article (for a discussion, see Datteri, 2021a). What really matters here is to note that this inferential step, and the corresponding justification, are not required in classical biorobotics, which is based on a totally different experimental procedure. Classical biorobotics does not raise this methodological issue [but it raises other methodological issues, widely discussed in Tamburrini and Datteri (2005) and Datteri and Tamburrini (2007)].

Both branches of biorobotics evoke questions about biomimicry: how similar should the robot be to its living counterpart, from a morphological and behavioral point of view, for the study to genuinely contribute to the life and social sciences? This question has different facets in the two fields and is to be addressed through different routes. Classical biorobotics typically adopts the synthetic method^1^. As discussed above, this method involves the building of a robot R which implements a mechanism *Mech* which, in turn, is hypothesized to govern the behavior of the living system L under investigation. R can be sensibly used to test *Mech* only if the former correctly implements the latter. But one thing is to require that R correctly implement *Mech*, another thing is to require that R be similar to the target system. And the second assumption is *not* made in the synthetic method. Indeed, the synthetic method has often led the experimenters to reject the hypothesis that *Mech* governs the behavior of the target system (an example being the study on bat echolocation discussed above). In those cases, R has genuinely contributed to scientific research—by providing empirical reasons to reject a hypothesis—even though it was not similar to L (because it implemented a biologically implausible mechanism). To sum up, what really matters in classical biorobotics is whether the robot correctly implements the hypothesized biological or cognitive mechanism, regardless of whether it is similar to the target system or not. The biomimicry question admits of a different answer in interactive biorobotics. There, it is legitimate to demand that the robot R be similar to the modeled system L, at least in the relevant morphological or behavioral respects. If such similarity obtains, L*’s reactions to R may be taken as an empirical basis to theorize on the reaction that L* would produce interacting with L, i.e., to justify the inferential step outlined in the paragraph above. This issue has been extensively discussed in Datteri (2021a).

To sum up. A methodological analysis of biorobotics may disclose the full range of uses of robots as experimental tools in the life and social sciences and pave the way for a discussion of the methodological issues distinctively arising in its branches. Distinguishing classical from interactive biorobotics may also enable one to acknowledge that such diverse research fields as rSCN and ethorobotics share common methodological features. The following sections will discuss how the interactive approach reconstructed here is adopted in rSCN (section “Bridging Humanoid Robotics and Social Cognitive Neuroscience”) and ethorobotics (section “Ethorobotics and Animal-Robot Interaction”).

## Bridging Humanoid Robotics and Social Cognitive Neuroscience

### Methodological Novelty

In 1950, Alan Turing proposed a test of “artificial intelligence” based on the ability to mimic human conversation called the “imitation game” (Turing, 1950). In the original version, a conversational artificial agent passes the Turing Test if a person interacting with it, through exchanges of written text (amounting to what we would today call an online chat), fails to recognize, when being asked explicitly, its artificial nature. It therefore requires (1) a human judge to discuss in writing with an unknown agent, and then (2) to determine, in a forced choice, whether this agent was a human or an automatic text synthesizer. In other words, an “artificial intelligence” is a machine devoid of any intentionality, thoughts, history, i.e., the internal mental states supposedly required for human communication, capable of holding a realistic human-like conversation even when being probed by a real human.

If one takes a reverse perspective, from the human instead of the machine point of view, “Computing Machinery and Intelligence” is particularly clever in how it proposes an operationalized perspective on what human intelligence is^2^. Yet such a definition is in conflict with current perspectives in social cognitive neurosciences, that emphasize hidden internal states, in contrast to observable behaviors, as defining features of human cognition. The original proposal also has a number of practical issues, most of them already considered by Alan Turing. First, such an interaction is relatively far from a natural social interaction given that the human conversant knows that the other agent is likely to be artificial and is asked to make a forced dichotomous choice. When the other agent is a human, both interlocutors know that they are “playing a game.” Neither situation happens in real life. Also, this test does not determine whether the machine behaves “intelligently,” a descriptor with too loose a meaning, torn between folk psychology, the meaning of IQ tests and the social ability under scrutiny, but whether it behaves as a human being would if it were asked the same questions in the same context of an “imitation game.” Other important limitations are the reliance on a simple linguistic vector, written text, and its corollary, the hidden position of the conversers: in humans, face-to-face—or at least voice-to-voice—interactions are the hallmark of social functioning. While these limitations are acceptable given that, on the one hand, machines don’t need embodiment to produce intelligible meaning, and, on the other hand, such impoverished exchanges indeed exist in humans, historically in the form of epistolary correspondence and more recently with emails or, even more relevant and more recent, online chats, the proposed approach remains reductive to investigate the scientific bases of human social interactions and their relevance to understand their human-machine counterparts.

Nevertheless, understanding, from a social cognitive neuroscience point of view, how artificial agents are perceived is an extremely timely question considering the contemporary incentive to introduce them in human everyday environments. Telephones’ voicebots and online virtual assistants are already largely present, in-home voice assistants entered households more recently, and it is expected that embodied humanoid robots should be the next step in putting humans in contact with “artificial intelligences”^3^. Therefore, rather than their purported “intelligence,” the key question to answer is how humans will consider and behave toward human-like artificial entities.

Is it possible to re-think the “Imitation game,” so as not to evaluate the machine’s “intelligence,” but its acceptance as a social partner one wants to interact with, something that can be called its social competence? One possibility could be to have people face an embodied robot, interact with it and evaluate to which extent their behavior mimics the behavior they have when facing fellow humans. As in the movie “Ex machina” (Garland, 2014), the robot’s face being perfectly human in all regards while many other cues point to an artificial agent, the imitation would somehow be reversed from the original test: assessed here is whether we “imitate ourselves” when interacting with an agent clearly identified as artificial—in other word, do we produce the same behaviors when facing an artificial agent resembling a human being than if it were a real human being. Going one step further, one can ask whether we use the same cognitive mechanisms and respective physiological underpinnings when interacting with human and human-like artificial agents. Although such a test seems to come straight out of science fiction, social cognitive neuroscience, and in particular the second-person neuroscience framework (Schilbach et al., 2013), provide a perfect point of view to investigate the behavioral and physiological mechanisms involved in Human-Human vs. Human-Robot interactions (Chaminade and Kawato, 2011; Chaminade, 2017; Rauchbauer et al., 2019). In addition this proposal isn’t completely new, as Android Science, introduced in particular by Karl McDorman and Hiroshi Ishiguro in the mid 2000 (MacDorman and Ishiguro, 2006), already considered using androids to study human cognition given their resemblance with humans (in shape and behavior) coupled with full reproducibility. But the current proposal of going further than the synthetic method goes further, as well as it gets rid of certain limitations of android science. Not only android science considered robots only as exact imitations of humans, in shape and behaviors, while we consider in the current proposal that that the artificial nature of the robot is an asset instead of a hindrance, it neglected something that appears clearly now, more than 15 years later, namely that such devices weren’t, and still aren’t, available. Limitations such as the uncanny valley, that too human-like imperfect robotic devices are repulsive instead of attractive, still appear very relevant despite decades of research and developments in humanoid robotics.

In the next section we provide examples of investigations on how interacting with natural or artificial agents, not androids, elicits different effects, both at the behavioral and physiological levels, on cognitive mechanisms pertaining to social cognition. Insights from this line of studies are twofold. Firstly, it helps address the open question whether artificial anthropomorphic agents should elicit the same effects as humans to be socially competent. Secondly, it informs us about which mechanisms involved in social cognition are influenced by the nature vs. the appearance and behavior of an interacting agent, akin to top-down vs. bottom-up processing. A simple example to illustrate this dichotomy is the case of artificial speech synthesizer of telephone robots: it is not because the source isn’t a real human that we don’t understand what it says. Actually, as for any heard speech, its understanding is irresistible. But on the other hand, philosopher Daniel Dennett argued that we don’t adopt an intentional stance, we don’t attribute mental states (Dennett, 1987), when interacting with a robot given it is devoid of intentions. This influence of the anthropomorphism of humanoid robots is challenging but goes beyond the current contribution; a recent discussion can be found in (Spatola and Chaminade, international journal of human—computer studies, bioXriv).

### Research Opportunities

#### Observing Humanoid Robots’ Actions

The Action Observation System (OAS) is a network of brain areas involved in perceiving and understanding human actions. Interestingly, two components of the OAS react differently when actions are depicted by a humanoid robot. Observing a robot executing human actions significantly increases the activity in occipital cortices, the component of the AOS associated with the processing of visual information, compared to the same actions executed by humans (Perani et al., 2001), independently of whether the action perceived needs to be understood or not (Chaminade et al., 2010). As a decrease of activity would be expected for unnatural stimuli if processing relied on a “matching to template” mechanism, the occipital action observation network rather appears to react as an error detector, with increased response to artificial agent’s actions because they are very different—in both shape and dynamics—than real actions. Such an effect can be compared to the fact that responses in the face fusiform area increase with the distance of individual faces from an average, prototypical, face (Loffler et al., 2005). This is a general mechanism, as the same is observed in auditory areas responding to voices: the further the distance from an average, prototypical voice, the more active these areas are (Latinus et al., 2013). Altogether, these results indicate that cortical areas involved in recognizing visual or auditory objects on the basis of sensory information areas are involved in a purely perceptual, or bottom-up, processing.

Another component of the AOS comprises motor areas of the posterior frontal cortex (premotor cortex). The automatic response of brain motor control systems caused by the perception of another’s actions is called “motor resonance.” In the same way that we cannot refrain from accessing the meaning of speech when we perceive it, we cannot refrain from activating our motor control systems when observing actions. Motor resonance can be measured as the influence that observing someone else’s actions has on the actions executed by the observer, an effect called motor interference. Indeed, motor interference is found, but reduced, when we observe the actions of a robot that has an overall human shape but clearly mechanical constituents (Oztop et al., 2005), while it does not if the robot is reduced to an industrial manipulator arm (Kilner et al., 2003; Oztop et al., 2005). At the neural level, response in the left inferior frontal gyrus, a premotor area involved in motor resonance, is reduced when the action is depicted by a robot instead of a human (Chaminade et al., 2010). Hence the mechanism involved in motor resonance is different from what we discussed in posterior sensory areas. Instead of indicating dissimilarity, increased activity indicates a matching to reality (see also Perani et al., 2001). For this reason, it has been argued for some time that this would be an important metric of how we relate with artificial agents (Chaminade and Cheng, 2009). Furthermore, in contrast to posterior areas, its response is modulated by the task of the observer. If forced to consider the robot’s movements as human actions (by asking them to rate “the emotions” vs. “the motion” of the robot presented on the screen), activity in premotor areas is significantly increased to reach the level of the activity in response to the human depicting the same emotions, while response to human emotions isn’t affected by the instruction. Therefore, results suggest reduction of motor resonance when observing humanoid robots can be contracted by instructing participants to see its movement as actions. A very similar conclusion has been drawn using a different approach, where the instruction to see the robot movement as actions primed robot anthropomorphism (Spatola and Chaminade, international journal of human—computer studies, bioRxiv). Human-robot interaction of the OAS confirm that the occipitotemporal action observation network responds, in a hard way, to the distance between the sensory prototypes and incoming information, with increased response when observing robots (vs. humans) executing movements, while the motor resonance network acts as a mirror, with reduced activity when the agent is a robot compared to a human, but sensitive to instruction, and possibly related to anthropomorphism of the robot.

#### Interacting With a Humanoid Robot

More recent work focused on real interactions with, and not mere observation of, humanoid robots. An fMRI study used a fully controlled stone-paper-scissors game as support of the interaction (Chaminade et al., 2012). Three brain regions were found to be significantly less responsive when humans (believed they) interacted with a robot, compared with another human: the medial prefrontal cortex and right temporoparietal junction (TPJ), both involved in mentalizing, and the hypothalamus, involved in social reward through the release of oxytocin. The significantly reduced response of the medial prefrontal cortex and right TPJ was also found using the prisoner’s dilemma as support of the interaction (Krach et al., 2008). In both cases, robots were used to manipulate the context of the social interaction, but the interaction itself remained fully controlled using well-established procedures from experimental social neuroscience. Therefore in these studies, it is the belief about the nature of the agent, rather than the agent itself, that explains differences in brain activity.

**FIGURE 3:**
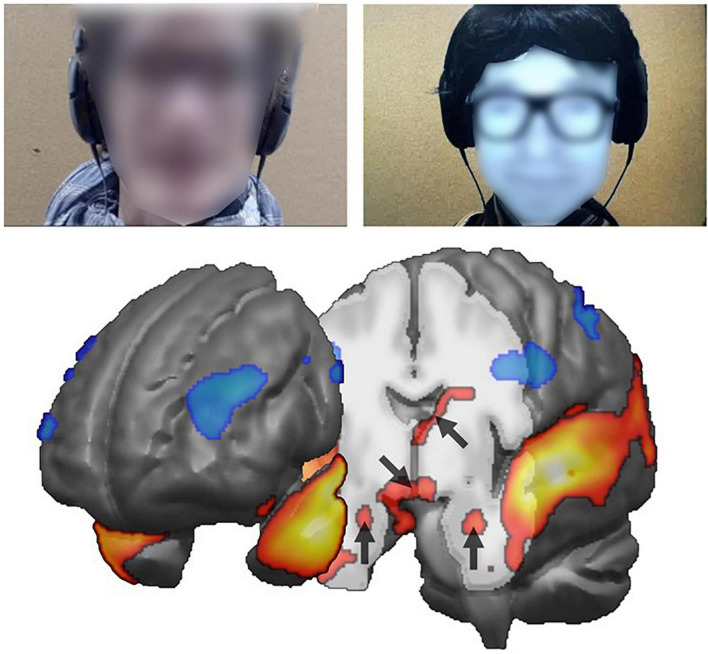
Comparing conversation with human vs. robot (**top**: extract from video) yields increased activity in subcortical structures, namely (arrows on the brain cut-off below, from **top** to **bottom**) the right caudate nucleus, the hypothalamus and the amygdala bilaterally.

A major step forward was the design of a truly interactive scenario. Participants were scanned with fMRI while they were discussing naturally either with a confederate of the experimenter, or with a robotic conversational head. Unbeknownst to them, the robotic device was controlled by the confederate using a Wizard of Oz procedure: participants always interacted with the same individual, but believed they were interacting with an autonomous agent. Results showed that areas involved in mentalizing (TPJ), emotions (amygdala) and social motivation (hypothalamus) were significantly more activated in human-human than in human-robot interactions (Figure 3). These results confirmed that participants adopt a different stance, including a reduced social motivation, when interacting with the artificial compared to the natural agent. These findings support the proposal that the activity in key brain structures can be used to assess implicitly the social competence of robotic designs along various dimensions of social cognition.

Further analysis of this corpus reveals that three key regions for autonomic processing of human emotions react differently to the happiness expressed by the conversational partner. In the insula and hypothalamus in particular, the positive correlation between the BOLD signal and the level of happiness expressed by the conversational agent is significant for the human partner, but absent when the interlocutor is the robot. If confirmed with a more emotionally expressive robot, this finding suggests that core autonomic systems used for social bonding only respond to human agents. Research with other categories of agents, such as robots with different embodiments, but also animals, as well as people from very different cultural backgrounds, would help prolong the current investigation on the mechanisms involved in human social cognition.

### Technological and Theoretical Challenges

As realistic android robots are emerging and the research in artificial intelligence promises to provide naturalistic conversational agents, some of the questions raised by the “Turing Test” are still contemporary: do we know whether an agent is natural or artificial? Can we, how can we, but also why should we want to know? And if we are to interact with such artificial beings, what features do we expect from them? In the previous section, it is proposed that local brain activity can provide information about cognitive mechanisms differing between human-human and human-robot interactions along pertinent dimensions of social interactions such as mentalizing and motivational processes. Using this information to describe human-human and human-robot interactions in a social cognitive neuroscience framework is fundamental to understanding what a likeable agent is, and how we consider artificial agents as interacting partners, and therefore holds the potential of complementing the Imitative Game in the social domain.

Our next challenge is far more complex. We’ve seen in the previous section that the effects on some mechanisms involved in social interactions are not static, but malleable under the effect of the context. What is unclear is how time, or experience, will influence the results as robots become increasingly present in our surroundings. Though hazardous, a comparison can be made with the animalization of native African at the time of slavery. The point here is not to draw a parallel with the nature of these individuals—clearly and undoubtedly humans, which is not the case for robots—but with the way they were perceived and conceived by the occidental, white population; a view that lasted for centuries and also took centuries to disappear (and still shows reminiscences today). If humans can dehumanize their fellows based on their perceived “otherness”^4^, can we imagine they can also humanize artifacts based on similarity with humans? While this can seem a philosophical, as well as politically incorrect, question, it raises doubts on whether current findings on the social competence of artificial agents are universal, or merely reflect a snapshot at a specific time in human cultural evolution, in which robots, given their recent history, remaining clumsiness and perception as mere mechanical artifacts, are far from having reached a stable societal representation (the one required in Android Science). Animal rights activism is a very contemporary example of what could happen in the future for robots, and the attribution of a citizenship to the gynoid robot Sophia in 2017, though highly anecdotal, was certainly thought as a precursor of things to come. As the “father” of Sophia puts it, “I think we should see the future with respect for all sentient beings and that will include machines^5^.” Addressing this issue scientifically is far-fetched but not insurmountable, and can teach us a lot on the flexibility of our mental representations pertaining to social interactions. A flexibility that has to be considered through the lens of cultural evolution, certainly a hallmark of human history.

## Ethorobotics and Animal-Robot Interaction

### Methodological Novelty

Research on animal–robot interaction, promoted by the high impact on society that robotics has made in the last decades (Yang et al., 2018; Romano et al., 2019), is rapidly growing. Robots are serving as advanced allies in investigating phenomena of behavioral adaptation, due to their high degree of manipulability and the possibility of controlling their position in the environment. They also allow one to perform highly standardized and reproducible experiments for the study of cognitive processes, such as perception, learning, memory and decision making.

In animal-robot interaction contexts, biomimetic agents are perceived as natural heterospecifics or conspecifics by animals, creating biohybrid dynamic systems where robots detect, communicate and interact with the animals, triggering specific neuro-behavioral responses, and adapting their behavior in function of the animal’s behavior (Cianca et al., 2013; Romano et al., 2017; Bierbach et al., 2018; Karakaya et al., 2020). This paradigm shift in the study of animal behavior—called “ethorobotics,” see Table 2-lies at the interface of ethology and robotics and has many potential applications in different areas including the control of animal populations in agriculture, the improvement of animal farming conditions, as well as in preserving wildlife.

The use of interactive artificial agents to study animal intelligence relies on multiple disciplines, such as biomimetics, robotics, machine learning, biosystems engineering, neuroethology, and more. Animals are elective model organisms for developing new methods in the aforementioned fields, thanks to their ability to learn and remember, their inter-individual differences, their adaptability to various experimental settings. These abilities can be exploited to face primary challenges in robotics, especially when artificial agents interact with abiotic and biotic factors, that also include interactions with humans, in the real world. It should also be noted that the field of ethorobotics is informing study of human-robot interaction too, emphasizing that social robots should have functions, as well as behaviors, and cognitive abilities fitting their specific environment. So, a robot endowed with social competence can produce meaningful and efficient interaction with humans independently of its human-like appearance (Miklósi et al., 2017).

Ethorobotics has several important objectives, including the improvement of animal wellness and environmental sustainability through mitigation of human activities on ecosystems. From an engineering point of view, animal-robot interaction can enable the creation of distributed hybrid networks of agents composed of animals and robots, bringing new emerging cognitive and physical capabilities to current bio-inspired robotic systems. And, more crucially for the purposes of the present paper, ethorobotics can importantly contribute to the study of animal behavior and cognition.

### Research Opportunities

Indeed, one of the most interesting goals of contemporary research on animal-robot interaction is the study of animals’ neuroethological and cognitive mechanisms. This goal is pursued in ethorobotics by exploring methodologies allowing living organisms to interact with robots in a natural way. Ethology is a scientific discipline devoted to the study of animal and human behavior in natural environments (Curtis and Houpt, 1983; McFarland, 1993; Lorenz, 2013). Research on interactive robots involves the study of organism-organism and organism-robot interaction by ethologists, as well as their cooperation in the design and modeling of robots’ morphology and behavior, to obtain functional artificial agents performing and fitting in the complex animal environment. In addition, interactive robots can be used to validate *in silico* systems, establishing open/closed-loop interactions with animals, and to manipulate specific behavioral displays “on demand” in animals.

Several stimuli have been traditionally used in ethology. Living subjects have been extensively used in many studies (Myrberg and Thresher, 1974; Willmott and Foster, 1995; Rowland, 1999), as they reflect the natural behavior of a species, generating realistic hypotheses that are typically tested by correlation analysis (Rowland, 1999). Although the use of freely interacting subjects provides the most natural social cues, it is very difficult to manipulate specific behavioral displays that can affect the response of focal subjects. Invasive approaches (e.g., freezing, surgical manipulation, pharmacological agents, etc.), can modify certain features of the live stimuli, but they are not ethically acceptable and should be permanently avoided, in order to comply with recent guidelines on animal behavior research (ASAB/ABS, 2020). Mirrors have long been used too in ethological studies to stimulate focal subjects (Leitão et al., 2019; Zablocki-Thomas et al., 2021) especially to visually duplicate conspecific features. However, the use of mirror has been often criticized due to the fact that it immediately reproduces feedback from the subject’s response, resulting in response rhythms that significantly differ from natural interactions. The use of static dummies and decoys with morphological features imitating conspecifics or heterospecifics has a respected tradition. Dummies can range from simple 2D silhouettes to 3D casted objects precisely reproducing visual features of the model species (Rowland, 1999; Tryjanowski et al., 2018). They are particularly powerful as far as static cues such as shape, size, posture and livery are concerned. However, the major limitation of dummies is their lower effectiveness in evoking response from focal subjects compared to living animals, also due to the difficulty in reproducing the movement of living systems. Playing back recorded sequences of behaviors is an additional method to provide stimuli during behavioral experiments (Rowland, 1999; Hirskyj-Douglas and Read, 2016; Chouinard-Thuly et al., 2017). Video playbacks permit the control and modification of visual features to simulate dynamic processes. However, these videos use imaging systems based on human perception, and cannot be equally perceived by species with a different spectral sensitivity, and flicker fusion rate. Also, video playbacks, as for mirrors, cut out the 3D exploration, as well as the tactile perception of the stimulus provided.

Robotics applied to ethological studies, obviously, offers capabilities extending far beyond the previously mentioned methods, allowing one to create biomimetic artificial agents performing complex behaviors that require sophisticated locomotion patterns or coordinated moving (Butail et al., 2014; Phamduy et al., 2014; Abdai et al., 2018; Bierbach et al., 2020; Romano and Stefanini, 2021a), and producing several kind of signals (Partan et al., 2009; Polverino et al., 2019; Romano and Stefanini, 2021b). Biomimetic agents can be fully controlled, allowing one to test all subjects with an identical set of cues. So, different responses from individuals are related to the intrinsic differences of focal animals and not to mutual influences between focal and stimulus individuals (Bierbach et al., 2021). Also, animal-robot interaction offers benefits as far as the welfare of the tested animals is concerned. In particular, the use of robots in ethological studies promotes the 3Rs Principle (Russell and Burch, 1959; Tannenbaum and Bennett, 2015), currently representing the gold standard for animal protection in laboratory conditions. The 3Rs Principle is based on the reduction of the number of individuals used in the experiments, the refinement of methods decreasing stress for animals, and wherever possible, the replacement of animals in tests by using alternative methods. Robots are particularly suitable for fulfilling the 3Rs Principle when studying aggressive behavior among conspecifics or predator-prey interaction between heterospecifics.

Furthermore, beside new knowledge, animal-robot interactive systems can produce a notable socio-economic impact on our daily lives, as well as on the influences of humans on the environment.

### Technological and Theoretical Challenges

Even though animal-robot interactive systems and ethorobotics have a huge scientific and technological potential, still, few research pathways have been explored. There is a growing interest in the use of robots in ethology. However, further efforts are needed to face several issues. A major challenge is connected to developing biomimetic robots able to adapt and negotiate with unstructured environments, to be released in natural habitats for interaction with animals. Also, energy harvesting and autonomy are crucial aspects that should be considered for long-lasting interactions in the real world. In addition, the development of robots which are effectively accepted as conspecifics by animals still represents a challenge that needs to be further addressed to boost the biomimetic design of interactive artificial agents. Furthermore, these mechatronic systems are often expensive and require dedicated expertise for their operation. As a result, still few research groups, with a multidisciplinary team, can use this advanced technology. So, it is crucial to promote worldwide collaborations between scientists with different backgrounds, due to the potential future impact that this field will have on the study of cognition and behavioral ecology.

## Taking Stock: Interactive Robots for the Study of Animal Behavior and Cognition

Robot-inspired social cognitive neuroscience (rSCN, section “Bridging Humanoid Robotics and Social Cognitive Neuroscience”) and ethorobotics (section “Ethorobotics and Animal-Robot Interaction”) involve building and experimenting with very different kinds of robots (humanoid and animaloid, respectively). Both fields introduce methodological novelties and open distinctive research opportunities for the study of the behavior and cognition of human and non-human animals, and give rise to distinctive technological and theoretical challenges. Despite their *prima facie* differences, they both exemplify *epistemic* uses of interactive robots, which are used as scientific tools for understanding animal behavior and cognition. Moreover, in this article it has been argued that rSCN and ethorobotics share a common methodological structure, which was illustrated in section “Introduction.” In both research fields, indeed, one builds a robot which may model a living system L, but is used to study the behavior and cognition of another living system L*. This strikingly differs from the “synthetic method” which has dominated cybernetic and biorobotic approaches to the study of animal behavior until recently.

The methodological structure outlined in section “Introduction” and exemplified by reference to the research fields discussed here may admit variants, some of which may still have to be investigated and instantiated. For example, recall that the classical biorobotics approach is comparative: the behavior of the robot is compared with the behavior of the modeled system in appropriate experimental conditions. The interactive methodology is not comparative *in the same sense* in which classical biorobotics is. In the vast majority of the ethorobotic studies carried out so far, for example, one learns something about the behavior and cognition of the system L* by analyzing its reactions to the stimuli exerted by the robot, with no comparison whatsoever between the behavior of L* and the behavior of the robot. To be sure, these studies often involve control groups in which the system L* interacts with robots differing in some respects from one another. But this kind of comparison is different from the comparative step involved in classical biorobotics, where *the behavior of the robot is compared with the behavior of the living system modeled by the robot*. A type of comparison which is more akin to classical biorobotics would consist in comparing the reactions of the system L* to the robot R with the reactions of the same system L* to the living system L modeled by the robot, or, more shortly, in comparing the dynamics of robot-animal (R-L*) interaction with the dynamics of animal-animal (L-L*) interaction. This sort of comparison has been relatively unexplored in ethorobotics, while it is made in many social cognitive neuroscience studies as illustrated in section “Bridging Humanoid Robotics and Social Cognitive Neuroscience,” where human reactions to robots are compared with human reactions to other human beings. Another comparative variant of the interactive strategy, envisaged in Datteri (2021b), would be to compare the dynamics of robot-animal (R-L*) interaction with the dynamics of interaction between the robot and *a robotic model of the focal system*. This variant might be of interest for studying the internal mechanisms guiding the behavior of the focal systems.

The considerations made in this article are mainly methodological. Reconstructing, and illustrating with examples, the rational structure of emerging approaches to the study of living systems may provide a basis to identify and discuss their methodological and theoretical complexities, some of which were considered before. Monitoring, understanding, and finally overcoming these complexities is of the utmost importance for placing robot-supported interactive approaches to the study of social behavior on firm methodological grounds. Collaboration between scientists, engineers, and philosophers of science—distinctively devoted to the study of the conceptual and rational foundations of scientific research—is essential to push classical and interactive forms of biorobotics to higher levels of methodological maturity.

## Author Contributions

ED wrote sections “Introduction” and “Taking Stock: Interactive Robots for the Study of Animal Behavior and Cognition.” TC wrote section “Bridging Humanoid Robotics and Social Cognitive Neuroscience.” DR wrote section “Ethorobotics and Animal-Robot Interaction.” All authors have made equal and substantial contributions to the conception and design of the study, the drafting and revision of it, and the final approval of the submitted version.

## Conflict of Interest

The authors declare that the research was conducted in the absence of any commercial or financial relationships that could be construed as a potential conflict of interest.

## Publisher’s Note

All claims expressed in this article are solely those of the authors and do not necessarily represent those of their affiliated organizations, or those of the publisher, the editors and the reviewers. Any product that may be evaluated in this article, or claim that may be made by its manufacturer, is not guaranteed or endorsed by the publisher.

## Footnotes

1 As pointed out in Datteri (2021a), prediction-oriented biorobotic studies do not conform to the synthetic method, while model-oriented studies do. However, the number of model-oriented studies published in the literature significantly surpasses the number of prediction-oriented studies. For this reason, even though in principle classical biorobotics needs not adopt the synthetic method, it is reasonable to say that it typically does.

2 Note, though, that some limitations should be taken into consideration, if only that all humans are far from being capable of using written language, and would thus lack “intelligence” if the definition was considered strictly.

3 Note that the meaning of “intelligence” in “artificial intelligence” can be very remote from the one defined by Alan Turing’s “Imitation game”; while it is very interesting that the main acceptance of the former, that one could roughly understand as generalized machine learning, is likely to be involved in the later, understood as reproducing human-like, in particular social, behaviors, in order to interact naturally with humans, these two acceptations of the word intelligence do not overlap.

4 Clearly “otherness” is a percept depending on the environment and past history in which it is made. Consider the thought experiment: if one lives his own life only surrounded by robots or non-human animals, wouldn’t humans be the strange, unusual, and maybe repulsive, agents?

5
https://www.cnbc.com/2017/12/05/hanson-robotics-ceo-sophia-the-robot-an-advocate-for-womens-rights.html
